# Draft Genome Sequences of Four Plant Probiotic *Bacillus* Strains

**DOI:** 10.1128/genomeA.00358-16

**Published:** 2016-05-12

**Authors:** Haeyoung Jeong, Seung-Hwan Park, Soo-Keun Choi

**Affiliations:** a Super-Bacteria Research Center, Korea Research Institute of Bioscience and Biotechnology (KRIBB), Daejeon, Republic of Korea; b Biosystems and Bioengineering Program, University of Science and Technology (UST), Daejeon, Republic of Korea

## Abstract

Here, we report the whole-genome sequences of four *Bacillus* strains that exhibit plant probiotic activities. Three of them are the type strains of *Bacillus endophyticus*, “*Bacillus gaemokensis*,” and *Bacillus trypoxylicola*, and the other, *Bacillus* sp. strain KCTC 13219, should be reclassified into a species belonging to the genus *Lysinibacillus*.

## GENOME ANNOUNCEMENT

*Bacillus* is one of the most extensively studied microorganisms among plant growth-promoting rhizobacteria (PGPR). Owing to the production of various antibiotics and the formation of stress-resistant endospores, *Bacillus* strains are more amenable to the formulation of commercial products and have been widely used as biocontrol agents (1). Induced systemic resistance (ISR) elicited by *Bacillus* strains has been recently recognized as one of the key mechanisms by which crops can protect themselves against phytopathogens with the aid of PGPR (2, 3).

The four *Bacillus* strains sequenced in this study, all purchased from the Korean Collection for Type Cultures (KCTC), were chosen based on the partial results of a systematic screening approach used to search for plant probiotic bacteria. The three type strains, *Bacillus endophyticus* KCTC 13922 (4), “*Bacillus gaemokensis*” KCTC 13318 (5), and *Bacillus trypoxylicola* KCTC 13244 (6), were shown to promote plant growth (*Arabidopsis thaliana*) by volatiles, whereas *Bacillus* sp. strain KCTC 13219 (=b04i-3) triggers ISR in *A. thaliana* against *Pectobacterium carotovorum* (our unpublished data). The type strains *B. gaemokensis* JCM 15801 and *B. trypoxylicola* NRBC 102646, obtained from the Japan Collection of Microorganisms (JCM) and the NITE Biological Resource Center (NBRC), respectively, were sequenced by other research groups and made available through accession numbers JOTM00000000 (7) and BCWA00000000 while this paper was being prepared.

Genome sequencing was carried out using the Illumina HiSeq 2000 platform at the National Instrument Center for Environmental Management at Seoul National University (Seoul, Republic of Korea). One hundred one-nucleotide paired reads produced from a library with a fragment size of ca. 500 bp were pretreated using Trimmomatic version 0.32 (8), and 600-Mb subsamples were randomly extracted from them. After k-mer-based error correction using SGA version 0.10.13 (9), *de novo* genome assembly was conducted using the A5-miseq pipeline (10). The assembled sequences were annotated using the RAST server (11) and Prokaryotic Genome Annotation Pipeline from the NCBI. Biosynthetic genes for secondary metabolites were predicted using antiSMASH 3.0 (12). The list of strains used in this study, the sequencing and assembly statistics, and the accession numbers are all provided in Table 1. Compared with the previously published records, the genome sequences of KCTC 13318 and KCTC 13244 obtained through this study were shown to be superior in terms of the assembly statistics.

KCTC 13219, isolated from Pu-erh tea, was tentatively named “*Bacillus nitroreducens*” by the submitter but did not lead to a proposal for a novel species (J. S. Lee, personal communication). Very recently, the species *Bacillus nitroreducens* sp. nov. was proposed for another unrelated bacterium (13). Phylogenetic analysis using the 16S rRNA sequence revealed that KCTC 13219 should be classified into the genus *Lysinibacillus* (99.93% similarity with “*Lysinibacillus fluoroglycofernilyticus*” cmg86^T^). The genome sequencing results will provide insight into the genomic basis of the versatile *Bacillus* strains and the interspecies interaction between soil bacteria and plants, which can lead to eco-friendly agricultural applications. Additional information is available at http://genoglobe.kr/kribb/four_bacillus_strains_2016.

### Nucleotide sequence accession numbers.

These whole-genome shotgun projects have been deposited at DDBJ/EMBL/GenBank, and the accession numbers for all four genome sequences are listed in Table 1.

**TABLE 1:** Summary of genome sequencing results

*Bacillus* species	Draft coverage (×)^a^	Genome size (bp)	No. of contigs	G+C content (%)	No. of secondary metabolite biosynthesis gene clusters^b^	Accession no.
*B. endophyticus* KCTC 13922^T^	741	5,121,484	42	36.50	11	LTAP00000000
*B. gaemokensis* KCTC 13318^T^	878	5,616,250	70	35.54	18	LTAQ00000000
*B. trypoxylicola* KCTC 13244^T^	9,024	4,347,941	40	35.73	6	LTAO00000000
*Bacillus* sp. KCTC 13219	1,244	3,853,058	37	38.31	3	LUFJ00000000

a Subsamples of 600 Mb were randomly taken from pretreated reads and assembled.

b Predicted by antiSMASH 3.0.
